# Individuals with ABO Groups Show Significant Differences in Levels of Circulating Biomarkers Related to Inflammation, Apoptosis, Endothelial Dysfunction, Tissue Remodeling and Neurodegeneration: A Pilot Study

**DOI:** 10.3390/diseases14060220

**Published:** 2026-06-19

**Authors:** Alessia Di Salvo, Chiara Motisi, Matteo Bulati, Letizia Scola, Carmela Rita Balistreri

**Affiliations:** 1 Cellular and Molecular and Clinical Pathological Laboratory, Department of Biomedicine, Neuroscience and Advanced Diagnostics (Bi.N.D.), University of Palermo, Corso Tukory 211, 90134 Palermo, Italy; 2 IRCCS ISMETT, 90127 Palermo, Italy

**Keywords:** ABO groups, circulating molecules, roles, clinical implications

## Abstract

Background and Objectives: Blood group antigens are well known for their importance in transfusion medicine and transplant compatibility; however, their biological role extends beyond these functions and includes associations with the risk of several diseases. In this study, we investigated the relationship between ABO blood groups and the circulating levels of 73 different molecules. Patients and Methods: Fifty-six healthy donors were enrolled, including 24 individuals with blood group O, 19 with blood group A, and 13 with blood group B. Blood samples were collected and analyzed in a single laboratory using Luminex fluorescent bead-based assay panels to determine the concentrations of 73 circulating molecules. Depending on data distribution, ANOVA or Kruskal–Wallis tests and Student’s *t*-test or Kolmogorov–Smirnov tests were applied to identify significant differences among groups. Associations were further assessed by binary logistic regression analysis. Results: Subjects with blood group A showed significantly higher circulating levels of IL-1R1, IL-13, IL-23, PDGF-BB, VEGF-A, VEGF-D, soluble VEGF-R2 (KDR), soluble VEGF-R3 (FLT-4), VLA-4, CD141, MMP-1, syndecan-1 (SDC-1), and mannose-binding lectin (MBL) compared with the other blood groups. In contrast, individuals with blood group B exhibited significantly higher levels of IL-22, IL-23, PDGF-BB, CD62P (P-selectin), and amyloid β1–42. Several significant associations were identified by logistic regression analysis. Conclusions: Our findings indicate that ABO blood groups are associated with distinct circulating molecular profiles, supporting the existence of biological differences that may contribute to variations in disease susceptibility among individuals with different blood types. Nevertheless, given the exploratory’s nature and limited sample size of this study, further investigations are required to validate these findings, confirm the observed associations, and clarify their potential clinical implications.

## 1. Introduction

ABO blood groups have traditionally been regarded as clinically relevant mainly in the context of transfusion medicine and transplant compatibility [1]. Nevertheless, accumulating evidence indicates that the biological significance of ABO antigens extends far beyond these classical applications. In recent years, several epidemiological and experimental studies have demonstrated associations between ABO blood groups and susceptibility to a variety of chronic conditions, including cardiovascular disease, venous thromboembolism, metabolic disorders, malignancies, and immune-mediated and inflammatory diseases [1,2]. These observations suggest that ABO blood group antigens may represent important determinants of interindividual variability in disease risk and progression.

The mechanisms underlying these associations are complex and multifactorial. Differences in ABO antigen expression have been shown to influence the plasma concentrations of several circulating proteins involved in hemostasis and thrombosis, most notably von Willebrand factor (vWF) and coagulation factor VIII, thereby contributing to differences in thrombotic risk among blood groups [1]. In addition, ABO antigens are widely expressed not only on erythrocytes but also on platelets, vascular endothelial cells, epithelial tissues, and in body secretions, suggesting that they may modulate multiple physiological and pathological processes [2,3]. Increasing evidence further indicates that ABO-dependent glycosylation patterns can affect cell–cell interactions, endothelial integrity, inflammatory signaling, and immune responses, ultimately influencing vascular homeostasis and tissue remodeling [3,4].

Beyond their role in coagulation, ABO blood groups have been associated with variations in circulating levels of adhesion molecules, cytokines, growth factors, and biomarkers of endothelial dysfunction and angiogenesis. Such molecules are critically involved in chronic low-grade inflammation, vascular remodeling, and cellular senescence, processes that are recognized as common pathogenic pathways underlying cardiovascular and neurodegenerative disorders [4,5,6]. Moreover, recent studies have suggested that blood group-related differences in inflammatory and immune mediators may contribute to the heterogeneity observed in individual susceptibility to chronic diseases and their clinical manifestations [5,6].

Despite the growing body of evidence linking ABO blood groups to disease risk, relatively few studies have comprehensively evaluated the relationship between ABO phenotypes and broad panels of circulating biomarkers encompassing inflammation, apoptosis, endothelial dysfunction, angiogenesis, extracellular matrix remodeling, and neurodegeneration. Therefore, the identification of specific molecular signatures associated with different blood groups may provide further insights into the biological mechanisms underlying these associations and contribute to the development of novel biomarkers for disease stratification and prevention.

To address this issue, we conducted a pilot study involving 56 healthy donors attending the Immunohematology and Transfusion Medicine Unit of the Paolo Giaccone University Hospital and affiliated AVIS centers. Using multiplex immunoassays, we investigated the association between ABO blood groups and a panel of 73 circulating molecules involved in inflammation, endothelial activation, angiogenesis, tissue remodeling, and neurodegenerative pathways, with the aim of identifying blood group-specific molecular profiles potentially associated with chronic disease susceptibility.

## 2. Patients and Methods

### 2.1. Population Selected in the Study

Fifty-six healthy donors were enrolled in the study. The study population had a mean age of 35 ± 0.2 years (range, 30–50 years) and consisted of 28 men and 28 women. Blood samples were collected at the Immunohematology and Transfusion Medicine Service of the Paolo Giaccone University Hospital and affiliated AVIS centers. According to the ABO blood group system, 24 participants belonged to blood group O, 19 to blood group A, and 13 to blood group B. Plasma samples were obtained at the time of donation and stored at −80 °C until analysis. The study protocol was approved by the Ethics Committee of the University Hospital of Palermo (approval number: 2024-UP-080424).

### 2.2. Quantification of Circulating Levels of 73 Molecules

Levels of 73 circulating biomarkers were measured by a single laboratory using Luminex fluorescent microsphere-based assay panels (ThermoFisher, Scientific, Waltham, MA, USA; Bender MedSystems GmbH, Campus Vienna Biocenter 2, A-1030 Vienna, Austria). Analyses were performed at a centralized laboratory according to the manufacturer’s protocol, and concentrations were determined with four- or five-parameter standard curves; samples were analyzed in duplicate to calculate mean measurements.

### 2.3. Statistical Analysis

For statistical analysis, data were analyzed using R software version 4.3.0 (The R Foundation) and GraphPad Prism 10 (GraphPad Software, La Jolla, CA, USA). All *p*-values were two-sided, and those less than 0.05 were considered statistically significant. For normally distributed quantitative variables, mean values with standard deviation were calculated. Differences between the three groups were assessed using one-way ANOVA or Kruskal–Wallis’s test, followed by Bonferroni correction, and the Kolmogorov–Smirnov test or Student’s *t*-test, as appropriate, to compare two groups. Binary logistic regression analysis was used to find the possible independent associations between age and levels of circulating molecules having significant differences between the three groups [7]. The Hosmer–Lemeshow test was used to check goodness-of-fit of the logistic regression.

## 3. Results

Statistical analyses revealed several significant differences among the study groups, as summarized in Table 1, Table 2 and Table 3. Subjects with blood group A exhibited significantly higher circulating levels of IL-1R1, IL-13, IL-23, PDGF-BB, VEGF-A, VEGF-D, soluble VEGF-R2 (KDR), soluble VEGF-R3 (FLT-4), VLA-4, thrombomodulin (CD141), MMP-1, syndecan-1 (SDC-1), and mannose-binding lectin (MBL) compared with the other blood groups (Table 1, Table 2 and Table 3). In contrast, individuals with blood group B showed significantly higher levels of IL-22, IL-23, PDGF-BB, CD62P (P-selectin), and amyloid β1–42 relative to the other groups (Table 1, Table 2 and Table 3).

Binary logistic regression analysis further demonstrated that blood groups A and B were associated with distinct, yet partially overlapping, biomarker profiles (Table 4). Group A was predominantly characterized by markers related to endothelial dysfunction, angiogenesis, and vascular remodeling, including VEGF-A, VEGF-D, PDGF-BB, and SDC-1. VEGF-A displayed one of the strongest associations (adjusted OR ≈ 2.4), suggesting a pronounced pro-angiogenic and endothelial activation profile. Conversely, blood group B was characterized by a pattern enriched in inflammatory cytokines (IL-22 and IL-23) and platelet activation markers (PDGF-BB and CD62P), consistent with a thrombo-inflammatory phenotype. IL-22 emerged as the strongest predictor in this group (OR up to 2), supporting its potential involvement in immune-mediated mechanisms.

Despite these differences, both blood groups shared common pathways related to chronic inflammation and vascular remodeling, processes that are known to contribute to the development of chronic disorders, including cardiovascular and neurodegenerative diseases. Overall, these findings suggest that ABO blood group status is associated with specific circulating molecular signatures that may partly underlie differences in susceptibility to chronic diseases.

## 4. Discussion

Several noteworthy findings emerged from the present pilot study, providing further evidence that ABO blood groups are associated with distinct circulating molecular signatures. These observations support the growing concept that ABO antigens represent not only determinants of transfusion compatibility, but also important modulators of immune, vascular, and metabolic pathways involved in the pathogenesis of chronic diseases [1,2,3,4,8]. Although the relatively small sample size warrants cautious interpretation, the molecular differences observed among blood groups suggest the existence of blood group-specific biological phenotypes.

Individuals with blood group A exhibited significantly higher circulating concentrations of IL-13 and IL-23, two cytokines that play pivotal roles in immune regulation and chronic inflammation. IL-13, a member of the T helper 2 (Th2) cytokine family, exerts pleiotropic effects on B lymphocytes, eosinophils, basophils, macrophages, fibroblasts, endothelial cells, and epithelial cells. Beyond its well-established involvement in allergic responses, IL-13 contributes to tissue remodeling, fibrosis, and the development of autoimmune diseases through the modulation of humoral immunity and inflammatory signaling pathways [9,10]. Interestingly, epidemiological studies have reported associations between ABO blood groups and inflammatory and immune-mediated disorders, suggesting that enhanced Th2 polarization may represent one of the underlying mechanisms [2].

Similarly, IL-23, a heterodimeric cytokine belonging to the IL-12 family, represents a central regulator of the IL-23/IL-17 axis and has emerged as a key mediator linking innate and adaptive immunity. Increased IL-23 signaling has been implicated in psoriasis, inflammatory bowel disease, rheumatoid arthritis, and tumor-associated inflammation, as well as in several hematological and solid malignancies [11,12,13]. The higher IL-23 levels observed in subjects with blood group A may therefore reflect a pro-inflammatory phenotype characterized by sustained activation of immune pathways associated with chronic diseases.

Another remarkable finding was the significantly elevated levels of VEGF-A, VEGF-D, and PDGF-BB detected in individuals with blood group A. These growth factors are essential mediators of angiogenesis and vascular remodeling and are critically involved in tissue repair, inflammation, and cancer progression. Increasing evidence indicates that inflammatory cytokines, particularly the IL-23/IL-17 axis, interact with VEGF-dependent pathways to promote pathological angiogenesis and support tumor growth and metastatic dissemination [12,13,14]. In this context, the coexistence of increased IL-23 and VEGF family members observed in blood group A suggests the presence of a coordinated inflammatory and pro-angiogenic profile. Notably, therapeutic interventions targeting IL-23 pathways have shown remarkable efficacy in psoriasis and are currently being explored in oncology because of their immunomodulatory effects [13,15].

Parallel to these findings, PDGF-BB represents one of the major regulators of vascular maturation and fibroblast activation. Dysregulated PDGF signaling is increasingly recognized as a hallmark of tumor angiogenesis and fibrosis and contributes to enhanced VEGF expression, tumor cell proliferation, and metastatic progression [14,16]. In agreement with this hypothesis, individuals with blood group A also displayed significantly higher levels of matrix metalloproteinase-1 (MMP-1), an enzyme involved in extracellular matrix degradation and tissue remodeling. Elevated MMP-1 concentrations have been associated with invasive tumor behavior and poor prognosis in several malignancies, supporting the notion that blood group A may be characterized by a molecular profile favoring angiogenesis and extracellular matrix turnover [17,18,19].

A further interesting observation was the increased concentration of thrombomodulin (CD141) and syndecan-1 (SDC-1), two transmembrane proteoglycans predominantly expressed by endothelial cells. Their soluble forms are generated following proteolytic shedding and are currently regarded as sensitive biomarkers of endothelial injury and glycocalyx degradation. Elevated circulating levels of soluble thrombomodulin and syndecan-1 have been described in cardiovascular diseases, sepsis, diabetes, chronic inflammatory conditions, and viral infections [20,21,22,23]. Since endothelial dysfunction represents a common pathogenic denominator linking thrombosis, inflammation, and vascular disease, the higher levels of these markers detected in blood group A may partly explain the increased risk of ischemic cardiovascular disease and thromboembolic complications previously reported in these individuals [3,24,25]. Moreover, these observations may provide additional mechanistic insights into the increased susceptibility of blood group A subjects to severe infectious diseases, including COVID-19 [5,26,27,28].

Interestingly, individuals with blood group A also exhibited increased circulating concentrations of mannose-binding lectin (MBL), an essential component of innate immunity and complement activation. MBL plays a crucial role in host defense against bacterial and viral pathogens and has been implicated in susceptibility to infectious and inflammatory diseases [29]. Previous studies have highlighted a relationship between MBL activity and coronavirus infections, including SARS-CoV-1, suggesting that alterations in lectin-mediated immunity may contribute to disease severity [30]. The concomitant elevation of MBL and endothelial markers in blood group A further supports the existence of a distinctive immunovascular phenotype.

Subjects belonging to blood group B displayed a distinct pattern characterized by significantly higher levels of IL-22, IL-23, PDGF-BB, CD62P (P-selectin), and amyloid β1–42. IL-22 is a cytokine involved in epithelial barrier homeostasis and tissue regeneration, but persistent activation of IL-22 signaling has been implicated in chronic inflammation, carcinogenesis, and immune-mediated diseases [31]. Together with IL-23, elevated IL-22 levels may indicate activation of the Th17 axis, which has been strongly associated with chronic inflammatory states and tumor progression [12].

In addition, blood group B subjects exhibited increased concentrations of soluble CD62P (P-selectin), a well-established marker of platelet activation and endothelial dysfunction. P-selectin mediates platelet–leukocyte interactions and contributes to thrombo-inflammatory processes that promote vascular injury, coagulation activation, and metastatic dissemination [32,33,34]. These findings are consistent with previous epidemiological observations reporting associations between blood group B and increased risks of gastric and other malignancies, as well as hematological neoplasms [4,35,36,37].

Another particularly intriguing finding was the higher concentration of amyloid β1–42 observed in blood group B individuals. Amyloid β peptides represent central mediators of neurodegenerative processes and are considered hallmark biomarkers of Alzheimer’s disease and other forms of dementia [38]. Increasing evidence indicates that vascular dysfunction and chronic inflammation may contribute to amyloid deposition and neurodegeneration [21,38]. Interestingly, previous studies have suggested associations between ABO blood groups and cognitive impairment and neurodegenerative disorders, raising the possibility that ABO-related mechanisms may influence neurodegenerative pathways [6,39].

Overall, the present findings support the existence of blood group-specific molecular signatures characterized by differential activation of inflammatory, endothelial, angiogenic, and neurodegenerative pathways. Such signatures may contribute to the heterogeneous susceptibility to chronic diseases observed among individuals with different ABO phenotypes.

An additional aspect deserving consideration concerns the central role of the ABO locus in protein glycosylation. The ABO gene encodes glycosyltransferases responsible for the addition of terminal carbohydrate residues to glycoproteins and glycolipids, thereby influencing protein folding, stability, receptor interactions, and clearance [40,41]. Consequently, ABO-dependent glycosylation patterns can modulate multiple biological processes, including coagulation, endothelial activation, leukocyte adhesion, inflammatory signaling, and angiogenesis. Genome-wide association studies have demonstrated that genetic variation within the ABO locus influences the circulating concentrations of numerous proteins, including soluble E-selectin, ICAM-1, vascular adhesion molecules, and several inflammatory mediators [32,33,42,43,44,45]. Furthermore, altered glycosylation patterns may regulate endothelial glycocalyx integrity, growth factor activity, and leukocyte trafficking, thereby contributing to vascular remodeling and chronic inflammation [21,22,46].

Taken together, these observations suggest that ABO blood groups may represent genetically determined modulators of systemic biological networks rather than simple erythrocyte antigens. The molecular profiles identified in the present study may therefore reflect distinct immunometabolic and vascular phenotypes that contribute to the variable susceptibility to cardiovascular diseases, cancer, autoimmune disorders, neurodegenerative diseases, and infectious diseases consistently associated with specific ABO blood groups [1,2,3,4,5,6,8,40,42]. Nevertheless, given the exploratory nature of this study and the limited number of participants, larger multicenter investigations integrating proteomic, glycomic, and genomic approaches are required to validate these findings and to clarify the clinical implications of ABO-associated molecular signatures.

## 5. Conclusions

The relatively small sample size represents an important limitation of the present study and may restrict the statistical power and generalizability of the findings. Therefore, the results should be interpreted with caution and considered exploratory. Nevertheless, the present data consistently indicate that ABO blood groups are associated with distinct circulating molecular signatures characterized by differential expression of biomarkers involved in inflammation, endothelial dysfunction, angiogenesis, tissue remodeling, platelet activation, and neurodegenerative pathways.

These observations agree with the growing body of evidence supporting a broader biological role of ABO antigens beyond their traditional significance in transfusion medicine and transplantation [32,43]. Indeed, the molecular profiles identified in this study suggest that blood groups A and B may represent distinct immunovascular phenotypes, potentially contributing to the heterogeneous susceptibility to chronic diseases reported among individuals with different ABO blood groups.

Although no causal inferences can be drawn from this pilot study, the observed associations provide additional biological plausibility to previously reported links between ABO blood groups and cardiovascular, inflammatory, neoplastic, neurodegenerative, and infectious disorders. Furthermore, the promising discriminatory performance observed in ROC analyses highlights the potential utility of integrated biomarker signatures for characterizing ABO-related phenotypes.

Overall, these findings support the concept that ABO blood groups may act as genetically determined modulators of systemic inflammatory and vascular networks rather than merely as erythrocyte antigens. Future large-scale, multicenter studies integrating proteomic, glycomic, genomic, and longitudinal clinical data are warranted to validate these results and to determine whether ABO-associated molecular signatures may have diagnostic, prognostic, or therapeutic implications in precision medicine.

## Figures and Tables

**Table 1:** Plasma levels of circulating cytokines and chemokines according to ABO blood groups.

Molecule	A Group (n = 19)	B Group (n = 13)	O Group (n = 24)	ANOVA *p*-Value	Significant Pairwise Comparisons
Cytokines					
IL-1α	0.5 ± 0.6	0.3 ± 0.3	1.3 ± 2.8	0.21	-
IL-1β	11 ± 7.2	10 ± 7	10.4 ± 6	0.94	-
IL-6	26.4 ± 25	37.3 ± 27	36 ± 23	0.35	-
IL-4	17 ± 7.3	15.1 ± 8	14 ± 8	0.45	-
IL-10	2.4 ± 1.4	3.3 ± 2	3.2 ± 2	0.18	-
IL-1R1	441 ± 242	183.3 ± 131.4	284 ± 158	0.001	A vs. B (<0.001); A vs. O (<0.005)
IL-13	8.5 ± 5	4.4 ± 3.4	5 ± 3.3	0.0061	A vs. B (<0.05); A vs. O (<0.05)
TNF-α	6.2 ± 4	6.3 ± 4	6 ± 3.4	0.97	-
IFN-γ	3 ± 2	2.4 ± 2	3 ± 2	0.93	-
IFN-α	1 ± 0.6	1 ± 0.6	1 ± 0.6	0.55	-
IL-2	14 ± 8	18 ± 9.4	19 ± 8	0.19	-
IL-12p70	0.5 ± 0.4	1 ± 0.4	1 ± 0.4	0.44	-
IL-15	14.1 ± 9	18 ± 9	16.4 ± 7.4	0.44	-
IL-17A	7.2 ± 4.3	10 ± 5.3	9 ± 5	0.23	-
IL-18	38 ± 20.4	35 ± 17.3	40 ± 18	0.74	-
IL-21	8 ± 6.2	9 ± 5.3	10 ± 9	0.68	-
IL-22	121.4 ± 42.3	37 ± 10.2	116 ± 40.4	<0.0001	A vs. B (<0.001); B vs. O (<0.001)
IL-23	14 ± 8	5.4 ± 4	13 ± 9	0.0065	A vs. B (<0.01); B vs. O (<0.05)
IL-27	33 ± 13	30 ± 15.1	31 ± 16	0.85	-
IL-31	9.1 ± 6	8 ± 5	8 ± 6	0.73	-
TNF-β	6.2 ± 4	4 ± 3	5 ± 3	0.19	-
IL-5	72 ± 50	94.2 ± 51	95 ± 52	0.29	-
IL-7	2 ± 1.2	2.3 ± 2	3 ± 2	0.26	-
GM-CSF	45.4 ± 30.4	50.2 ± 22.2	58 ± 24.2	0.29	-
LIF	6 ± 2	7 ± 2.3	6 ± 2	0.55	-
Chemokines					
IL-8 (CXCL8)	2.2 ± 2	3.4 ± 2	4 ± 2.4	0.08	-
GROα (CXCL1)	6 ± 3	7 ± 3.3	6 ± 2	0.43	-
MCP-1 (CCL2)	37 ± 28	42.4 ± 22	33 ± 18	0.47	-
MIP-1α (CCL3)	1.3 ± 1	2 ± 1	2 ± 1.1	0.40	-
MIP-1β (CCL4)	45 ± 16	43 ± 16.4	40 ± 16	0.60	-
RANTES (CCL5)	36.2 ± 12.2	30.2 ± 14	27.4 ± 14	0.10	-
IP-10 (CXCL10)	12 ± 4	12.1 ± 6	11.3 ± 6.3	0.89	-
Eotaxin (CCL11)	15.1 ± 7.2	15.1 ± 6.3	13.13 ± 6	0.54	-
SDF-1α	567 ± 425	311 ± 153	377.4 ± 279.2	0.057	-

**Table 2:** Plasma levels of growth factors and endothelial dysfunction molecules according to ABO blood groups.

		Growth Factors			
Molecule	A Group (n = 19)	B Group (n = 13)	O Group (n = 24)	ANOVA *p*-Value	Significant Pairwise Comparisons
BDNF	41 ± 23.4	55 ± 33.2	50.3 ± 25	0.31	-
EGF	16.3 ± 10.4	10 ± 6	13 ± 10	0.11	-
HGF	58 ± 30.4	53 ± 28	55 ± 31.2	0.89	-
FGF-2	6 ± 5	9 ± 7	8.4 ± 5.3	0.14	-
PDGF-BB	317.2 ± 285	713 ± 360	431 ± 265.2	0.0019	A vs. B (<0.01); B vs. O (<0.05)
PlGF-1	7 ± 5	8 ± 3	6.5 ± 5	0.64	-
SCF	4 ± 3.5	3 ± 2	3 ± 3.2	0.73	-
**Endothelial Dysfunction Molecules**					
**Molecule**	**A Group (n = 19)**	**B Group (n = 13)**	**O Group (n = 24)**	**ANOVA *p*-Value**	**Significant Pairwise Comparisons**
VEGF-A	251 ± 194	92 ± 54	136 ± 96.3	0.0028	A vs. B (<0.01); A vs. O (<0.05)
VEGF-D	173.3 ± 162.3	51 ± 19.2	91.2 ± 73.3	0.0057	A vs. B (<0.01); A vs. O (<0.05)
VEGF-R2	5121 ± 1584	3632 ± 1142	4131 ± 1104	0.0057	A vs. B (<0.01); A vs. O (<0.05)
VEGF-R3	21,730.4 ± 8608.3	12,864.2 ± 5312.4	16,295.4 ± 7208	0.0044	A vs. B (<0.01)
VLA-4	2340.4 ± 1380	1110 ± 912.4	1212 ± 559.4	0.0005	A vs. B (<0.01); A vs. O (<0.01)
Angiopoietin-1	520 ± 412.2	312 ± 220	437.3 ± 385	0.29	-
CD31 (PECAM-1)	13,046 ± 3033.4	12,032 ± 3408	12,068 ± 3727.4	0.59	-
CD62P	46,560 ± 32,745	25,436 ± 9438	46,109 ± 21,067	0.029	B vs. O (<0.05)
CD62E	14,882.2 ± 17,872.2	6485 ± 3697	11,222 ± 4604	0.11	-
Thrombomodulin	534 ± 295.3	266.4 ± 123.3	323 ± 155	0.0009	A vs. B (<0.01); A vs. O (<0.01)
MMP-1	119 ± 105	25.3 ± 19	55 ± 50	0.001	A vs. B (<0.01); A vs. O (<0.05)
Syndecan-1	208 ± 142	87 ± 45	120 ± 68.4	0.002	A vs. B (<0.01); A vs. O (<0.05)

**Table 3:** Plasma levels of apoptotic molecules, metalloproteinases and molecules involved in neurodegeneration according to ABO blood groups.

Type	Molecule	Group A (Mean ± SD)	Group B (Mean ± SD)	Group 0 (Mean ± SD)	*p*-Value
Apoptosis	AXL	11,195.2 ± 3380	12,106.4 ± 3305	11,124.2 ± 2317	0.59
	CALR (CRT)	6030.2 ± 2134.2	5463 ± 1628	5476 ± 1945	0.59
	CD36	2623 ± 1078	2432.4 ± 760.4	2344.4 ± 803	0.59
	Gas6	2755 ± 794	2439 ± 697	2345 ± 575.2	0.14
	LOX-1	37 ± 8	36.3 ± 16	36 ± 13	0.96
	MBL	13,544 ± 8618.2	9182 ± 8682	5813.2 ± 4261	0.0035
	Mer (MERTK)	1776.2 ± 577	1555 ± 334	1555 ± 462	0.27
	PAI-1 (Serpin)	1697.4 ± 631.2	1651.4 ± 842	1762 ± 770.2	0.90
	RAGE	44 ± 28.2	34.01 ± 11	38 ± 25.4	0.49
	TYRO3	703.2 ± 186	602 ± 126	611 ± 142	0.10
	uPAR	1569 ± 467.4	1447.4 ± 336.3	1441.3 ± 359.2	0.53
Metalloproteinases	MMP-2	21.2 ± 19	13.4 ± 8	15 ± 8	0.17
	MMP-3	44 ± 20	49.4 ± 28	40.4 ± 27	0.58
	MMP-9	49 ± 21	50 ± 20	55 ± 27.4	0.68
Neurodegeneration	Amyloid β1–42	0.6 ± 0.13	0.7 ± 0.3	0.5 ± 0.1	0.004
	Neurogranin (NRGN)	325.2 ± 384.2	161 ± 129	226 ± 191.2	0.21
	NCAM-1	22,933 ± 13,019	27,879.2 ± 16,298	21,165 ± 12,299	0.36
	Kallikrein-6 (KLK6)	684 ± 353	730 ± 354	652 ± 230.4	0.76
	Tau (Total)	63.2 ± 43.3	58.3 ± 30.3	63 ± 34.2	0.91
	Tau (pT181)	4 ± 2.2	3 ± 2	3 ± 2	0.24

**Table 4:** Univariate and multivariate regression analysis of demographic factors and circulating molecules associated with A and B groups in the study population.

Group	Variable	Unadjusted OR (95% CI)	*p*-Value	Adjusted OR (95% CI)	*p*-Value
A	Age	1.04 (1.03–1.09)	0.03	1.05 (0.92–1.03)	0.02
	IL-1R1	1.05 (0.91–1.9)	0.01	1.21 (0.89–1.2)	0.047
	IL-13	1.29 (0.94–1.4)	0.03	1.54 (1.3–2.3)	0.02
	IL-23	1.49 (0.70–1.8)	0.04	1.01 (1.39–2.2)	0.047
	PDGF-BB	1.68 (1.7–2.5)	0.01	1.8 (1.7–2.8)	0.02
	VEGF-A	2.6 (1.1–2.7)	0.01	2.4 (1.6–3.1)	0.03
	VEGF-D	1.3 (0.94–1.4)	0.04	1.54 (1.3–2.3)	0.041
	sVEGF-R2 (KDR)	1.5 (0.79–1.2)	0.035	1.9 (1.39–2.4)	0.037
	sVEGF-R3 (FLT-4)	1.49 (0.70–1.8)	0.04	-	-
	Thrombomodulin (CD141)	1.5 (1.4–2.8)	0.01	1.2 (1.4–2.9)	0.02
	MMP-1	1.8 (1.5–1.9)	0.037	1.04 (1.3–2.9)	0.04
	SDC-1	1.68 (1.7–2.5)	0.01	1.8 (1.7–2.8)	0.02
	MBL	1.05 (0.91–1.9)	0.01	1.21 (0.89–1.2)	0.047
B	Age	1.03 (0.93–1.8)	0.027	1.21 (0.89–1.2)	0.03
	IL-22	1.8 (1.7–2.5)	0.01	2 (1.7–2.8)	0.02
	IL-23	1.3 (0.94–1.4)	0.04	1.54 (1.3–2.3)	0.036
	PDGF-BB	1.87 (1.9–2.8)	0.02	1.9 (1.8–2.9)	0.038
	CD62P	1.5 (0.89–2)	0.04	-	-
	Amyloid β1–42	1.04 (1.03–1.09)	0.048	-	-

Adjusted for age, IL-1R1, IL-13, IL-22, IL-23, PDGF-BB, VEGF-A, VEGF-D, sVEGF-R2 (KDR), sVEGF-R3 (FLT-4), Thrombomodulin (CD141), MMP-1, SDC-1, MBL, CD62P (P-selectin), and Amyloid β1–42. The Hosmer–Lemeshow test statistic was 7.01 (df = 7.9, *p* = 0.53), which revealed good model fit.

## Data Availability

The data presented in this study are contained within the manuscript and are available from the corresponding author upon reasonable request.
